# Determining a future policy focus to support antimicrobial stewardship in community pharmacy: A modified Delphi study

**DOI:** 10.1016/j.rcsop.2024.100456

**Published:** 2024-05-23

**Authors:** Kathryn Lim, Elaine Lum, Anna Olsen, Lisa Nissen, Alex Broom, Holly Seale

**Affiliations:** a School of Population Health, University of New South Wales, Australia; b Duke-NUS Medical School, National University of Singapore, Singapore; c ANU College of Health and Medicine, Australian National University, Australia; d Centre for the Business and Economics of Health, The University of Queensland, Australia; e School of Social and Political Sciences, The University of Sydney, Australia

**Keywords:** Antimicrobial stewardship, Antimicrobial resistance, Pharmacy, Pharmacists, Qualitative research, Policy

## Abstract

**Background:**

Over the past decade, the pharmacy sector's policy, academic and professional spheres have increasingly drawn attention to the opportunities to better leverage the untapped potential of the community pharmacy sector in contributing to global efforts to reduce antimicrobial resistance (AMR). While efforts are in train, progress is slow.

**Objective:**

To draw insights from global experts in the field to identify a broad range of potential future policy directions to support community pharmacists' involvement in antimicrobial stewardship (AMS).

**Methods:**

A modified Delphi technique, comprising two survey rounds to build consensus amongst global community pharmacy sector stakeholders and opinion leaders. In Round 1, participants rated their level of agreement with 28 statements across the three domains of policy design, implementation design, and monitoring and evaluation. Participants were also invited to contribute feedback in Round 1, which was reflected as new statements (*n* = 10) in Round 2. In Round 2, participants were asked to re-assess Round 1 statements in view of the group consensus and to rate the new statements.

**Results:**

289 participants were invited to participate. 48/289 (17% response rate) completed Round 1, and 25/42 (60% response rate) completed Round 2. Consensus (defined as >70% agreement) was achieved for 79% (*n* = 30) of the statements across the three domains.

**Conclusion:**

Pharmacy sector experts agreed that acknowledging community pharmacists in AMS national action plans is an important component, signalling a recognition of the sector's contribution to whole-of-nation AMS efforts. Implementation components that achieved consensus reflect the profession's evolution to a professional service driven model, particularly in complementary AMS initiatives including infection prevention and control measures. Context-specific adjustments to support implementing these AMS measures will be required, in addition to striking the appropriate balance to support the pace of increased community pharmacists'involvement in AMS with building whole-of-profession buy-in.

## 1 Introduction

Primary health care plays a central role in supporting global efforts to reduce the rise of antimicrobial resistance (AMR) by contributing to antimicrobial stewardship (AMS) efforts.^1^ The World Health Organization's (WHO) Global Action Plan for AMR^2^ sets out the strategic objectives to tackling AMR globally which includes: effective antimicrobial surveillance, optimising antimicrobial use, improved infection prevention and control measures, global awareness campaigns, and increasing research and development into novel antimicrobials.^2^ Commonly underpinning these strategic objectives is the call for collective efforts across sectors, within and across nations, to address AMR – which is often codified in national actions plans to ensure context specific relevance.^2^^,^^3^ Despite this, national approaches to address AMR are frequently focused on dedicated efforts within the tertiary health care sector and in surveillance and monitoring activities.^1^ Directing AMS policies and practices at hospitals limits the potential for a whole-of-health system approach to tackling AMR, particularly as primary health care is a key access point for the community to antimicrobials.^1^

As primary health care providers, community pharmacists are underutilised in AMS efforts.^4^^,^^5^ Harnessing the full breadth of pharmacists' ability to contribute to optimising antimicrobial use is a view held within the pharmacy sector's policy, academic and professional spheres,^6^ and is promoted through the profession's peak organization's position statements on AMS^7^ and professional publications.^8^ These publications position the breadth of community pharmacists' AMS contribution within a ‘guardian and gatekeeper’^6^ framework – fulfilling an information broker role to promote public awareness and understanding of the need for optimal antimicrobial use; coupled with performing a triage function to support individuals with minor ailments to determine if a general practitioner consultation, and potential antimicrobial is required.^6^ This positioning likely reflects the nature of contemporary pharmacy practice, which has shifted beyond a process orientated service, to one that encompasses professional service delivery - evidenced most strongly in supportive AMS practices such as pharmacy-based vaccination services that have become a mainstay globally.^9^ Despite this messaging, AMS initiatives in community pharmacy^10^ are often focused on promoting the profession's ability to provide public education about AMR^11^ by uplifting community pharmacists' AMS awareness and knowledge. Amplifying community pharmacists' information-provision role, reinforced by a continued policy focus on primarily promoting AMS education and training for community pharmacists risks a narrow perception and engagement of the profession in contributing to whole-of-system AMS efforts.^12^

Constrained health system resources means that efforts to expand community pharmacists' involvement in AMS activities requires consideration of the relative merit of strategies or initiatives that will best achieve this aim. Adopting the view of the majority can inform where efforts should be prioritised; identification of which can occur using consensus based questions, a technique characteristic of the Delphi process.^13^ The Delphi technique is premised on the principle that increased accuracy of decisions can be achieved through structured stakeholder feedback over unstructured or individual views^14^ – an approach that was initially applied in military applications to support forecasting, which has since been adopted for use in other fields such as health sciences.^13^

The aim of this study was to draw insights from global experts in the field to identify a broad range of potential future policy directions to support community pharmacists' involvement in AMS. Potential policy directions identified could be used by policy makers in reviewing and rebalancing national AMS plans to include a greater focus on the contribution of primary health care, supported by harnessing the latent potential of community pharmacists.

## 2 Methods

This study used a modified survey Delphi technique^15^^,^^16^ to conduct a two-stage consensus building approach on potential policy options for community pharmacists' involvement in AMS. The structured questioning ‘rounds’ used in the Delphi technique enables consensus to be ascertained from subject matter experts.^16^ Increasingly, the Delphi approach has been used in pharmacy practice research to support the development of policies, guidelines, and performance indicators.^17^

### 2.1 Survey development

The governance framework for AMR detailed by Anderson and colleagues (2019)^3^ was used to broadly inform the design of the survey structure. As a conceptual model, the framework was designed to support policymakers to systematically navigate the process of designing, implementing, and monitoring their AMR National Action Plans (NAPs).^3^ Structuring the Delphi survey using these governance framework domains provided consideration of how the statements assessed by subject matter experts in this study could easily translate into how pharmacists' involvement could be articulated in AMR NAPs in the future. Not all the domains in the framework were reflected in the survey, as some did not have specific application to the pharmacy sector.

The survey statements were derived from previous studies conducted by members of the research team relating to the external^7^^,^^8^^,^^18^ and internal^6^ characterisations of the role of community pharmacists' in AMS. The survey statements reflected the research team's analysis of common key findings and proposed policy solutions from across the previous studies for testing with the expert panel through this study. This resulted in 28 pre-determined statements for testing through the Round 1 survey across three domains of 1) policy design, 2) implementation design, and 3) monitoring and evaluation. The subsequent survey (Round 2) built on Round 1 results, with the survey instrument presented in the supplementary file.

The survey was drafted by K.L. and improvements in the structure, readability and content validity of the statements included in the survey was achieved through an iterative process in consultation with the research team (H.S., E.L., L.N., A.O., and A.B.) who have a range of experience in AMS and AMR-related research, and clinical pharmacy.

### 2.2 Panel recruitment

Participants were recruited using a combined purposive and snowball sampling approach. This sought to ensure that invited participants met the inclusion criteria for the study: recognition as either being a community pharmacy sector stakeholder or opinion leader; aged 18 years and older; and able to complete the survey in English. Community pharmacy sector stakeholders or opinion leaders were defined as individuals in clinical practice, policy and/or program development, or research. Due to the breadth of the professionals invited to participate in the panel and recognising that individuals often hold multiple roles (for example, being in policy development and clinical practice), the research team decided not to analyse any data identified by profession.

Participants were sourced from publicly available information, including relevant pharmacy professional organisations such as the International Pharmaceutical Federation (FIP)’s Commission on AMR,^19^ and contributors and reviewers to key AMR related publications.^20^^,^^21^ Participants were sourced predominantly from high-income countries, such as the United Kingdom and the United States of America, as countries with comparable community pharmacy models to Australia as the survey questions were derived largely from Australian based studies. Invited participants were also encouraged to share the invitation with their professional networks based on the study's eligibility criteria.

For the second round of the Delphi, only participants who had taken part in the first round and indicated interest to participate in the subsequent round by sharing their email addresses were contacted. Up to two personalised reminders were sent to participants to maximise response rate. The research team took a pragmatic approach to determining the number of survey rounds, seeking to minimise participant burden recognising that individuals were not provided incentives to participate.

The Human Research Ethics Committee at the University of New South Wales granted approval (HC#230327) for this study.

### 2.3 Data collection

**Fig. 1:**
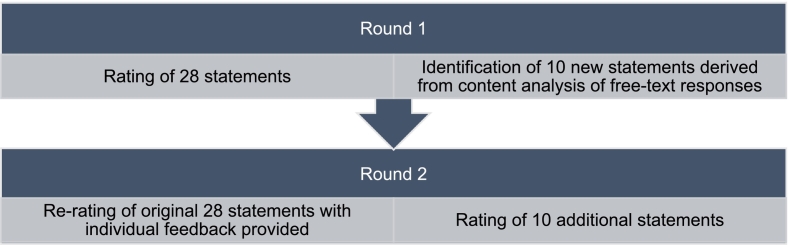
Flow diagram illustrating the two rounds of the Delphi study conducted.

The survey was conducted in two rounds (Fig. 1 below) between September 2023 and December 2023. Surveys were administered online using Qualtrics™ (Qualtrics, Provo, UT). Invitations to participate were sent via email, along with the Participant Information Statement which noted the anonymous and voluntary nature of participation. Completing the survey was taken as participants providing informed consent. Identifiable participant information was only accessed by the research team to administer the study, with anonymity maintained between participants.

#### 2.3.1 Delphi Round 1

In the first round of the modified Delphi, participants assessed their level of agreement against the 28 statements. Level of agreement was assessed using a 5-point Likert scale (‘strongly agree, agree, neither agree nor disagree, disagree, and strongly disagree’). For each domain, participants were also given the option to provide free-text comments to suggest amendments to the statements or additional statements for consideration within the domain assessed.

#### 2.3.2 Delphi Round 2

Data from round one were analysed using descriptive statistics (survey) and content analysis (free text). Consistent with maintaining anonymity between participants, agreement percentages for each round one statement were provided to individuals, along with their previous individual ratings. Additional statements derived from content analysis of the free text responses received in the previous round were generated into new survey questions for ranking (*n* = 10). Participants were asked to assess their level of agreement across the 38 statements using the same 5-point Likert scale in round one.

### 2.4 Data analysis

Responses were analysed following the completion of each survey round. Descriptive statistics, including frequency percentages were used to present the survey results. Data were collated in Microsoft Excel and imported to SPSS (IBM SPSS Statistics for Windows, Version 26.0. Armonk, NY, USA) for further analyses. Similar to other Delphi studies,^22^^,^^23^ consensus was considered met if the combined level of agreement of ‘strongly agree’/’agree’ was >70% across all participants for each statement. Content analysis^24^ was completed for the open-text comments received in round one. The analysis focused on identifying additional statements for testing in Round 2.

## 3 Results

Of the initial 289 participants invited to participate in the study, 48 completed Round 1. Of the 48 Round 1 participants, 42 indicated interest to participate in Round 2; 25 (60%) completed the subsequent survey. Experts involved were from across government, non-government and academic spheres and were predominantly located across Australia, Europe, and North America.

Table 1 below shows a summary of the Delphi statements grouped by the domains and sub-domains explored in the surveys. In Round 1, consensus (defined as the combined level of agreement of ‘strongly agree’/’agree’ >70%) was achieved for 82.1% (*n* = 23) of the 28 statements, with this decreasing in Round 2 to 78.9% (*n* = 30) of the 38 statements. Only three domains/sub-domains reached consensus across both rounds: policy design; infection prevention and control; and awareness.

**Table 1:** Summary of number of statements in each survey domain, including consensus ratings.

Survey domains and sub-domains	Number of statements in each domain and sub-domain		Proportion of statements where consensus⁎ was achieved (n)	
	Round 1	Round 2	Round 1	Round 2
Domain 1: Policy design	6	8	83.3% (5)	87.5% (7)
Domain 2 A: Implementation design - Antimicrobial stewardship	9	11	66.7% (6)	63.6% (7)
Domain 2B: Implementation design - Infection prevention and control	6	8	100.0% (6)	87.5% (7)
Domain 2C: Implementation design - Education	2	3	50.0% (1)	66.7% (2)
Domain 2D: Implementation design - Awareness	4	5	100.0% (4)	100.0% (5)
Domain 3: Monitoring and evaluation	1	3	100.0% (1)	66.7% (2)
Total	28	38	82.1% (23)	78.9% (30)

⁎ Defined as combined level of agreement of ‘strongly agree/’agree’ >70%.

Table 2 presents the level of consensus against each statement grouped by domain. Statements that achieved consensus in Round 1, also reached consensus in Round 2 (Table 2). For the Policy Design domain, 7 out of 8 statements at the end of Round 2 reached consensus; the statement relating to relative participation in AMS activities by hospital and community pharmacists did not reach consensus. Within the Implementation Design domain, consensus was achieved for all five statements relating to public awareness (100%). The sub-domain of antimicrobial stewardship had the highest number of statements which did not achieve consensus. The four statements that did not reach consensus in this sub-domain related to perceived familiarity with the term ‘AMS’, alternative phrasing for AMS, and approach to repeat antimicrobial prescriptions. Seven out of eight statements achieved consensus within the sub-domain of infection prevention and control (IPC) following Round 2, with the statement relating to discussing point of care diagnostic results with a prescriber not reaching consensus. The education sub-domain achieved consensus for two out of three statements following Round 2, with the statement relating to prioritising interprofessional skills and communication over AMS not achieving consensus. Within the Monitoring and Evaluation domain, the two statements that achieved consensus related to routine data collection and integrating data collection within existing workflows. The one statement within this domain that did not achieve consensus related to remuneration for data collection activities.

**Table 2:** Level of consensus to each statement in the surveys grouped by domain.

	**Round 1**			**Round 2**		
Statements	Level of consensus^a^ (%)	Median rating^b^	SD	Level of consensus^a^ (%)	Median rating^b^	SD
**Domain: Policy design**						
All Antimicrobial Resistance National Action Plans (NAPs) should include mention of the primary care sector's contribution.	**91.3%**	1	1.0	**92.0%**	1	1.0
Pharmacy should be explicitly referenced when primary care is described in NAPs.	**89.1%**	1	1.1	**96.0%**	1	0.9
When describing pharmacists' contribution to addressing AMR, the practice setting should also be specified (e.g. hospital, community).	**77.8%**	1	1.2	**96.0%**	1	0.9
The breadth of pharmacists' contribution to addressing AMR is linked to the practice setting.	**71.7%**	2	1.2	**72.0%**	2	1.1
Pharmacists in the hospital setting can participate in more antimicrobial stewardship activities compared to pharmacists in the community setting.	50.0%	3	1.4	48.0%	3	1.3
Representatives from the pharmacy sector should be clearly engaged in developing, revising, and evaluating AMR-NAPs.	**89.1%**	1	1.2	**92.0%**	1	1.0
Pharmacists work in multi-disciplinary health care teams and participate in AMS practices through their distinct roles in hospital and community settings which can vary across regions.	–	–	–	**84.0%**	1	1.0
NAPs should reference the need for collaboration between hospital and community pharmacists in supporting AMS.	–	–	–	**80.0%**	1	1.2

**Domain 2A: Implementation design – Antimicrobial stewardship**						
Antimicrobial stewardship is a familiar term to community pharmacists.	53.3%	2	1.1	48.0%	3	0.9
Antimicrobial stewardship should be discussed as part of pharmacists' role to promote the quality use of medicines.	**95.5%**	1	0.7	**100.0%**	1	0.5
The term ‘quality use of medicines’ should be used instead of ‘antimicrobial stewardship’ when discussing this topic with the community pharmacy sector.	17.8%	4	1.1	16.0%	4	1.0
Community pharmacists' key contribution to antimicrobial stewardship is limited to providing antimicrobial medicines information.	**80.0%**^c^	4	1.0	**92.0%**^c^	4	0.7
Community pharmacists' antimicrobial stewardship role is not limited to providing medicines information.	**84.1%**	1	0.9	**96.0%**	2	0.6
Key enablers, such as digital tools, should be designed and implemented to facilitate information transfer in primary care to promote multidisciplinary antimicrobial stewardship practice.	**95.6%**	2	0.7	**100.0%**	2	0.5
Funding should be provided to community pharmacies to implement antimicrobial stewardship programs.	**84.5%**	2	0.9	**88.0%**	2	0.6
Accreditation standards for community pharmacy should include evidentiary requirements for antimicrobial stewardship.	**75.6%**	2	0.9	**80.0%**	2	0.8
Community pharmacists should not dispense a repeat prescription for antibiotics unless clinical appropriateness can be determined.	68.8%	2	1.0	60.0%	2	0.9
Provision of clinical indication on antibiotic prescriptions should be mandatory to support community pharmacists to assess clinical appropriateness prior to dispensing.	–	–	–	**83.3%**	2	0.8
The term “quality use of antimicrobials” is an appropriate phrasing to use when discussing this topic with the community pharmacy sector.	–	–	–	40.0%	3	1.0

**Domain 2B: Implementation design - Infection prevention and control**						
Community pharmacists should be routinely provided diagnostic information to support antimicrobial stewardship considerations.	**82.2%**	2	0.9	**80.0%**	2	0.8
The public should be encouraged to seek community pharmacists' advice for minor ailments (e.g. cold and flu).	**84.4%**	1	0.8	**92.0%**	1	0.6
Clinical protocols for managing minor ailments in community pharmacy should be established.	**80.0%**	1	1.0	**96.0%**	1	0.7
Community pharmacists should be made aware of the link between infection prevention and control measures in supporting antimicrobial stewardship.	**88.9%**	1	0.8	**96.0%**	1	0.6
Community pharmacists should be encouraged to promote vaccination as part of their stewardship role.	**97.7%**	1	0.5	**100.0%**	1	0.5
Diagnostic tools, such as point of care testing, should be made available in community pharmacy.	**79.5%**	2	0.9	**80.0%**	2	1.0
Community pharmacists need to be adequately trained to appropriately use diagnostic tools, including understanding appropriate circumstances for use and limitations.	–	–	–	**88.0%**	1	0.7
If community pharmacists collect diagnostic results (such as through point of care testing), these results should be discussed with a patient's prescriber.				68%	1	1.0

**Domain 2C: Implementation design - Education**						
Antimicrobial stewardship should be a key professional competency for pharmacists.	**88.9%**	1	0.8	**92.0%**	1	0.7
Professional development on antimicrobial stewardship should prioritise developing communication and interprofessional skills over clinical knowledge.	48.9%	3	1.1	28%	3	1.2
Professional development on antimicrobial stewardship should ensure clinical knowledge and communication and interprofessional skills are equally developed.	–	–	–	**96.0%**	1	0.6

**Domain 2D: Implementation design - Public awareness**						
There is a need to increase awareness of community pharmacy's involvement in antimicrobial stewardship amongst health policy leaders.	**93.3%**	1	0.6	**96.0%**	1	0.6
There is a need to increase awareness of community pharmacy's involvement in antimicrobial stewardship amongst health professionals.	**93.3%**	1	0.7	**95.8%**	1	0.6
There is a need to increase awareness of community pharmacy's involvement in antimicrobial stewardship amongst the public.	**86.7%**	1	0.8	**91.7%**	1	0.7
Community pharmacists should be promoted as key information sources for the public about antimicrobials.	**84.5%**	1	0.9	**92.0%**	1	0.7
Collaborative efforts by all health professionals in addressing antimicrobial stewardship should be promoted.	–	–	–	**100.0%**	1	0.4

**Domain 3: Monitoring and evaluation**						
Data about pharmacy-based minor ailments services should be routinely collected and include information about patient outcomes.	**71.1%**	2	1.1	**76.0%**	2	0.9
Any monitoring and evaluation activities for antimicrobial stewardship in community pharmacy should be harmonised into existing workflows.	–	–	–	**88.0%**	2	0.7
Data collection activities relating to antimicrobial stewardship that are undertaken by community pharmacists should be remunerated.	–	–	–	64.0%	2	0.8

Note: Bold % indicates that consensus was achieved (combined level of agreement of ‘strongly agree’/’agree’ >70%).

a Level of consensus = % Strongly Agree + % Agree.

b Ratings were from 1 (strongly agree) to 5 (strongly disagree)

c Reverse coded.

Some participants who provided free-text comments described a potential ‘intent-action’ gap. A key theme related to contextual factors that may influence the feasibility of implementing the statements tested – with a spectrum of responses indicating that some participants were operating in contexts where these statements reflected their current state, while others reflected on these statements being ‘nice but almost unfeasible in my country’.

## 4 Discussion

Amongst pharmacy sector experts, consensus on 30 components for future policy consideration across the domains of policy design, implementation, and monitoring and evaluation to support community pharmacists' involvement in AMS was achieved. A summary of the broad potential future policy directions supported by panellists includes:

• Ensuring pharmacists are involved in, and contribute content to, the development and review of national action plans for addressing AMR to enable profession-specific and context-specific inclusions;
• Continuing to raise awareness amongst the public and health professionals of the role of community pharmacists in AMS, which includes leveraging their increasing involvement in infection prevention and control (IPC) activities as a complementary AMS function;
• Increasing AMS relevant data and monitoring activities in community pharmacy, which strikes an appropriate balance between breadth of data gathered and the related administrative burden.

### 4.1 Domain 1: Policy design

National action plans (NAPs) are the primary mechanism through which countries publicly set out their strategic priorities and actions to tackle AMR.^25^ Panellists reached consensus on the role of pharmacists in both the development process and indicative content relating to pharmacy that should be contained within NAPs. Though the sector has had representative input26., 27., 28 to key global AMR guidance, continuing pharmacist engagement with the development of NAPs can ensure a contextually relevant reflection of pharmacists' AMS involvement. This is critical as complex contextual factors such as social and political dimensions^29^ influence AMR development in different ways globally. Though the characterisation of pharmacists' AMS involvement is articulated in the profession's position statements,^7^ acknowledgement in a NAP can signal a recognition of the profession's contribution within a nation's holistic, multisectoral approach to addressing AMR which may drive action.^30^ The panel's consensus view on the need to reference primary care in NAPs reflects the World Health Organization's call for expansion of NAPs beyond a focus on tertiary settings to focus on primary and community care settings.^1^ Interestingly, the panellists' views that the distinct contributions of pharmacists from different clinical settings should be acknowledged. However, this focus on the differences, rather than the complementarities of pharmacists in addressing AMR through their respective clinical settings may continue to challenge the realisation of a “whole of profession” effort.^8^

### 4.2 Domain 2: Implementation design

Raising public awareness of the community pharmacy's contribution to AMS achieved 100% consensus amongst experts. However, experts did not reach consensus on whether the term “AMS” is familiar to community pharmacy despite previous research suggesting that community pharmacists are aware of AMS concepts.^31^ This may introduce tensions with how the sector's AMS contribution is broadly described, when the sector itself may not be familiar with the term. Experts were not able to also reach consensus on the appropriateness of promoting AMS within the context of delivering on a quality use of medicines agenda which may better resonate.^6^

Experts agreed that community pharmacists' AMS role was not limited to providing medicines information, with this supported by the high levels of consensus achieved regarding community pharmacists' promoting IPC activities. Ensuring pharmacists are aware of the link between AMS and IPC is a core domain of the WHO Competency Framework for Health Workers' Education and Training on AMR,^28^ and opportunities to promote this linkage should be leveraged. Community pharmacy led minor ailments schemes^12^ such as the United Kingdom's NHS Pharmacy First Service^32^ and Australia's NSW Pharmacy Trials^33^ where community pharmacists are able to provide over the counter antimicrobials under specific protocols for conditions such as uncomplicated urinary tract infections (UTIs) are an avenue to promote this linkage. This is key as these services increasingly become part of the profession's core business.^6^ Promoting community pharmacists' AMS role within their IPC related professional service offerings may also address any negative perceptions of community pharmacists' influence in shifting the dial on AMR.^34^

However, as health systems increasingly pivot to enable community pharmacists to adopt professional identities as clinicians providing clinical services, research has suggested that this may not be an identity uniformly shared by front-line pharmacists.^35^ Kellar and colleagues (2021)^35^ suggested that pharmacists hold strong identities as dispensers and patient counsellors rather than clinicians; with this dissonance a potential contributing factor to challenging wide-scale practice change. Implementing health system reform that enables community pharmacists to adopt a greater involvement in AMS, therefore, needs to be cognisant of the rate and scale of buy-in from community pharmacists to ensure any gap between expectations and reality of the profession's AMS involvement is minimised.

### 4.3 Domain 3: Monitoring and evaluation

Experts reached consensus on the need to monitor and collect pharmacy-based minor ailments scheme data – reflecting current practice of relevant initiatives underway.^32^^,^^33^ Most participants agreed that data collection activities should be harmonised within existing workflows, but consensus was not reached around renumerating pharmacists for this activity. Detecting patterns of antimicrobial consumption in the community is key to building a holistic understanding of antimicrobial use, but community-level data requires strengthening.^1^ Data collection activities should consider a bidirectional flow – that is, data entered into the system supports system surveillance, but also is shared back to a health professional to support quality improvement about their practice.^36^ This can include enhancing behaviour through positive feedback, as explored by Jones and colleagues (2019)^37^ through a quality improvement project that focused on a strengths-based approach to providing health professionals positive feedback on AMS practices. It resulted in some process improvements and decreased broad-spectrum antibiotic consumption. This approach may be explored with community pharmacists, using existing data sets such as those collected through pharmacy-based minor ailments schemes,^32^^,^^33^ as a first step to introducing data-driven feedback loops to support AMS.

### 4.4 Strengths and limitations

Gathering consensus through a network of AMS, AMR and community pharmacy experts through this Delphi study has enabled the research team to bring together a collection of potential policy options for the profession to pursue as it increases its visibility and contribution to AMS globally. It is acknowledged that the study's results are reflective of the composition of the panel, which may not be representative of their demography, knowledge, interests, and expertise. Recruitment of participants from predominantly high-income countries may also limit the generalisability of these findings, particularly within the context of implementation in low-and middle-income countries. However, the broad concepts of the statements that achieved consensus remain applicable and should be adapted to achieve a context-appropriate approach. Future studies may seek to build on these findings by testing and validating their context-specific relevance with region specific AMS and pharmacy experts. Participant self-selection based on the inclusion criteria which set out the parameters for expertise may also limit the generalisability of the results. However, as the initial recruitment list was drawn from professional organization information and relevant literature, this implies a degree of expertise in the field of pharmacy and AMS that would have satisfied the condition of expertise required to participate in this study.

Similar to other Delphi studies,^22^ the sample size achieved was above that of the appropriate sample despite reduced response rates across the rounds. No agreement currently exists regarding the optimal panel size or definition of small or large samples for Delphi studies.^38^ However, as suggested by Keeney and colleagues (2011),^38^ a smaller sample size (up to 15–20 participants) was considered an appropriate recruitment target given the relative homogeneity of the participants – with this being sufficient to infer generalisability and representativeness of the results.^38^

Further, due to involving an international panel and consideration of response burden, the modified Delphi was conducted using an online survey and limited to two rounds. Though further rounds may have yielded the opportunity to achieve definitive consensus on outstanding statements, these statements should not be disregarded as they may be of relevance within specific contexts.^39^

## 5 Conclusion

Better leveraging the untapped potential of the community pharmacy sector to contribute to global efforts in reducing AMR has drawn much attention over the past decade. While efforts have been made in Australia and internationally to include community pharmacists in AMR reducing efforts, progress is slow. This study shows that there is broad expert consensus on the key policy levers for engaging community pharmacists, highlighting that there is a will to adjust future policy directions. Balancing the pace of reform with ensuring whole-of-profession buy-in will be a critical success factor.

## CRediT authorship contribution statement

**Kathryn Lim:** Writing – review & editing, Writing – original draft, Methodology, Formal analysis, Conceptualization. **Elaine Lum:** Writing – review & editing, Methodology. **Anna Olsen:** Writing – review & editing, Supervision, Methodology. **Lisa Nissen:** Methodology. **Alex Broom:** Supervision. **Holly Seale:** Writing – review & editing, Supervision, Methodology.

## Declaration of competing interest

The authors declare that they have no known competing financial interests or personal relationships that could have appeared to influence the work reported in this paper.

## Appendix A Supplementary data

Survey - Determining a future policy focus to support antimicrobial stewardship in community pharmacy Image 1
