# Supplementary material for: Determining a future policy focus to support antimicrobial stewardship in community pharmacy: A modified Delphi study
Source: Explor Res Clin Soc Pharm. 2024 May 23;14:100456. doi: 10.1016/j.rcsop.2024.100456 (PMC11214505; doi:10.1016/j.rcsop.2024.100456)
Supplement: Supplementary file 1 — Survey - Determining a future policy focus to support antimicrobial stewardship in community pharmacy [file mmc1.docx]

**Supplementary File: Survey - Determining a future policy focus to support antimicrobial stewardship in community pharmacy**

**Round 2 Survey**

| **#** | **Status (from Round 1)** | **Statements** | **Response Option** |
| --- | --- | --- | --- |
| **Domain 1: Policy design** | | | |
| 1 | Re-rating | All Antimicrobial Resistance National Action Plans (NAPs) should include mention of the primary care sector’s contribution. | 5-point Likert scale (Strongly Disagree to Strongly Agree) |
| 2 | Re-rating | Pharmacy should be explicitly referenced when primary care is described in NAPs. | 5-point Likert scale (Strongly Disagree to Strongly Agree) |
| 3 | Re-rating | When describing pharmacists’ contribution to addressing AMR, the practice setting should also be specified (e.g. hospital, community). | 5-point Likert scale (Strongly Disagree to Strongly Agree) |
| 4 | Re-rating | The breadth of pharmacists’ contribution to addressing AMR is linked to the practice setting. | 5-point Likert scale (Strongly Disagree to Strongly Agree) |
| 5 | Re-rating | Pharmacists in the hospital setting can participate in more antimicrobial stewardship activities compared to pharmacists in the community setting. | 5-point Likert scale (Strongly Disagree to Strongly Agree) |
| 6 | Re-rating | Representatives from the pharmacy sector should be clearly engaged in developing, revising and evaluating AMR-NAPs. | 5-point Likert scale (Strongly Disagree to Strongly Agree) |
| 7 | New | Pharmacists work in multi-disciplinary health care teams and participate in AMS practices through their distinct roles in hospital and community settings which can vary across regions. | 5-point Likert scale (Strongly Disagree to Strongly Agree) |
| 8 | New | NAPs should reference the need for collaboration between hospital and community pharmacists in supporting AMS. | 5-point Likert scale (Strongly Disagree to Strongly Agree) |

| **Domain 2: Implementation design** | | | |
| --- | --- | --- | --- |
| **Domain 2A - Antimicrobial Stewardship** | | | |
| **#** | **Status (from Round 1)** | **Statements** | **Response options** |
| 9 | Re-rating | Antimicrobial stewardship is a familiar term to community pharmacists. | 5-point Likert scale (Strongly Disagree to Strongly Agree) |
| 10 | Re-rating | Antimicrobial stewardship should be discussed as part of pharmacists’ role to promote the quality use of medicines. | 5-point Likert scale (Strongly Disagree to Strongly Agree) |
| 11 | Re-rating | The term ‘quality use of medicines’ should be used instead of ‘antimicrobial stewardship’ when discussing this topic with the community pharmacy sector. | 5-point Likert scale (Strongly Disagree to Strongly Agree) |
| 12 | Re-rating | Community pharmacists’ key contribution to antimicrobial stewardship is limited to providing antimicrobial medicines information. | 5-point Likert scale (Strongly Disagree to Strongly Agree) |
| 13 | Re-rating | Community pharmacists’ antimicrobial stewardship role is not limited to providing medicines information. | 5-point Likert scale (Strongly Disagree to Strongly Agree) |
| 14 | Re-rating | Key enablers, such as digital tools, should be designed and implemented to facilitate information transfer in primary care to promote multidisciplinary antimicrobial stewardship practice. | 5-point Likert scale (Strongly Disagree to Strongly Agree) |
| 15 | Re-rating | Funding should be provided to community pharmacies to implement antimicrobial stewardship programs. | 5-point Likert scale (Strongly Disagree to Strongly Agree) |
| 16 | Re-rating | Accreditation standards for community pharmacy should include evidentiary requirements for antimicrobial stewardship. | 5-point Likert scale (Strongly Disagree to Strongly Agree) |
| 17 | Re-rating | Community pharmacists should not dispense a repeat prescription for antibiotics unless clinical appropriateness can be determined. | 5-point Likert scale (Strongly Disagree to Strongly Agree) |
| 18 | New | Provision of clinical indication on antibiotic prescriptions should be mandatory to support community pharmacists to assess clinical appropriateness prior to dispensing. | 5-point Likert scale (Strongly Disagree to Strongly Agree) |
| 19 | New | The term “quality use of antimicrobials” is an appropriate phrasing to use when discussing this topic with the community pharmacy sector. | 5-point Likert scale (Strongly Disagree to Strongly Agree) |

| **Domain 2B: Infection prevention and control** | | | |
| --- | --- | --- | --- |
| **#** | **Status (from Round 1)** | **Statements** | **Response options** |
| 20 | Re-rating | Community pharmacists should be routinely provided diagnostic information to support antimicrobial stewardship considerations. | 5-point Likert scale (Strongly Disagree to Strongly Agree) |
| 21 | Re-rating | The public should be encouraged to seek community pharmacists’ advice for minor ailments (e.g. cold and flu). | 5-point Likert scale (Strongly Disagree to Strongly Agree) |
| 22 | Re-rating | Clinical protocols for managing minor ailments in community pharmacy should be established. | 5-point Likert scale (Strongly Disagree to Strongly Agree) |
| 23 | Re-rating | Community pharmacists should be made aware of the link between infection prevention and control measures in supporting antimicrobial stewardship. | 5-point Likert scale (Strongly Disagree to Strongly Agree) |
| 24 | Re-rating | Community pharmacists should be encouraged to promote vaccination as part of their stewardship role. | 5-point Likert scale (Strongly Disagree to Strongly Agree) |
| 25 | Re-rating | Diagnostic tools, such as point of care testing, should be made available in community pharmacy. | 5-point Likert scale (Strongly Disagree to Strongly Agree) |
| 26 | New | Community pharmacists need to be adequately trained to appropriately use diagnostic tools, including understanding appropriate circumstances for use and limitations. | 5-point Likert scale (Strongly Disagree to Strongly Agree) |
| 27 | New | If community pharmacists collect diagnostic results (such as through point of care testing), these results should be discussed with a patient's prescriber. | 5-point Likert scale (Strongly Disagree to Strongly Agree) |

| **Domain 2C: Education** | | | |
| --- | --- | --- | --- |
| 28 | Re-rating | Antimicrobial stewardship should be a key professional competency for pharmacists. | 5-point Likert scale (Strongly Disagree to Strongly Agree) |
| 29 | Re-rating | Professional development on antimicrobial stewardship should prioritise developing communication and interprofessional skills over clinical knowledge. | 5-point Likert scale (Strongly Disagree to Strongly Agree) |
| 30 | New | Professional development on antimicrobial stewardship should ensure clinical knowledge and communication and interprofessional skills are equally developed. | 5-point Likert scale (Strongly Disagree to Strongly Agree) |

| **Domain 2D: Public awareness** | | | |
| --- | --- | --- | --- |
| 31 | Re-rating | There is a need to increase awareness of community pharmacy’s involvement in antimicrobial stewardship amongst health policy leaders. | 5-point Likert scale (Strongly Disagree to Strongly Agree) |
| 32 | Re-rating | There is a need to increase awareness of community pharmacy’s involvement in antimicrobial stewardship amongst health professionals. | 5-point Likert scale (Strongly Disagree to Strongly Agree) |
| 33 | Re-rating | There is a need to increase awareness of community pharmacy’s involvement in antimicrobial stewardship amongst the public. | 5-point Likert scale (Strongly Disagree to Strongly Agree) |
| 34 | Re-rating | Community pharmacists should be promoted as key information sources for the public about antimicrobials. | 5-point Likert scale (Strongly Disagree to Strongly Agree) |
| 35 | New | Collaborative efforts by all health professionals in addressing antimicrobial stewardship should be promoted. | 5-point Likert scale (Strongly Disagree to Strongly Agree) |

| **Domain 3: Monitoring and evaluation** | | | |
| --- | --- | --- | --- |
| 36 | Re-rating | Data about pharmacy-based minor ailments services should be routinely collected and include information about patient outcomes. | 5-point Likert scale (Strongly Disagree to Strongly Agree) |
| 37 | New | Any monitoring and evaluation activities for antimicrobial stewardship in community pharmacy should be harmonised into existing workflows. | 5-point Likert scale (Strongly Disagree to Strongly Agree) |
| 38 | New | Data collection activities relating to antimicrobial stewardship that are undertaken by community pharmacists should be remunerated. | 5-point Likert scale (Strongly Disagree to Strongly Agree) |
